# Relative Contributions of the Novel Diarylquinoline TBAJ-876 and its Active Metabolite to the Bactericidal Activity in a Murine Model of Tuberculosis

**DOI:** 10.1093/infdis/jiae332

**Published:** 2024-06-28

**Authors:** Saskia E Mudde, Nicole C Ammerman, Marian T ten Kate, Nader Fotouhi, Manisha U Lotlikar, Hannelore I Bax, Jurriaan E M de Steenwinkel

**Affiliations:** Department of Medical Microbiology and Infectious Diseases, Erasmus University Medical Center, Rotterdam, The Netherlands; Department of Medical Microbiology and Infectious Diseases, Erasmus University Medical Center, Rotterdam, The Netherlands; Department of Medical Microbiology and Infectious Diseases, Erasmus University Medical Center, Rotterdam, The Netherlands; Global Alliance for Tuberculosis Drug Development, New York, New York, USA; Global Alliance for Tuberculosis Drug Development, New York, New York, USA; Department of Medical Microbiology and Infectious Diseases, Erasmus University Medical Center, Rotterdam, The Netherlands; Department of Internal Medicine, Section of Infectious Diseases, Erasmus University Medical Center, Rotterdam, The Netherlands; Department of Medical Microbiology and Infectious Diseases, Erasmus University Medical Center, Rotterdam, The Netherlands

**Keywords:** tuberculosis, TBAJ-876, metabolite, bactericidal activity, mouse model

## Abstract

**Background:**

TBAJ-876 is a next-generation diarylquinoline. In vivo, diarylquinoline metabolites are formed with activity against *Mycobacterium tuberculosis*. Species-specific differences in parent drug-to-metabolite ratios might impact the translational value of animal model-based predictions. This study investigates the contribution of TBAJ-876 and its major active metabolite, TBAJ-876-M3 (M3), to the total bactericidal activity in a mouse tuberculosis model.

**Methods:**

In vitro activity of TBAJ-876 and M3 was investigated and compared to bedaquiline. Subsequently, a dose-response study was conducted in *M. tuberculosis*-infected BALB/c mice treated with TBAJ-876 (1.6/6.3/25 mg/kg) or M3 (3.1/12.5/50 mg/kg). Colony-forming units in the lungs and TBAJ-876 and M3 plasma concentrations were determined. M3's contribution to TBAJ-876's bactericidal activity was estimated based on M3 exposure following TBAJ-876 treatment and corresponding M3 activity observed in M3-treated animals.

**Results:**

TBAJ-876 and M3 demonstrated profound bactericidal activity. Lungs of mice treated for 4 weeks with 50 mg/kg M3 were culture negative. Following TBAJ-876 treatment, M3 exposures were 2.2 to 3.6-fold higher than for TBAJ-876. TBAJ-876 activity was substantially attributable to M3, given its high exposure and potent activity.

**Conclusions:**

These findings emphasize the need to consider metabolites and their potentially distinct exposure and activity profiles compared to parent drugs to enhance the translational value of mouse model-driven predictions.

## Teaser

The profound bactericidal activity of the new diarylquinoline TBAJ-876 in a tuberculosis mouse model was substantially attributable to in vivo formation of the highly active M3 metabolite, highlighting the need to consider metabolites when translating mouse model findings to humans.

Tuberculosis remains a major global health challenge [1], and the ongoing need to improve tuberculosis treatment outcomes has led to an expansion of the tuberculosis drug development pipeline. This resulted in the introduction of bedaquiline, a diarylquinoline that acts by inhibiting ATP synthase, which transformed the approach to treating drug-resistant tuberculosis. Multiple clinical trials demonstrated treatment success rates of approximately 90% when bedaquiline-based regimens were administered to drug-resistant tuberculosis patients [2–5]. This led to the World Health Organization endorsement of a 6-month treatment regimen including bedaquiline, pretomanid, and linezolid (BPaL), with or without moxifloxacin, for the treatment of drug-resistant tuberculosis [6].

While proven to be an effective drug, bedaquiline comes with limitations. Its affinity for cardiac potassium hERG channels can lead to QTc interval prolongation, with the risk of cardiac arrhythmias [7–10]. Bedaquiline is highly lipophilic, contributing to drug accumulation and a long half-life, which may lead to prolonged drug exposure after treatment cessation [8, 9]. There are concerns that lingering low plasma concentrations might lead to resistance development, putting the usability of bedaquiline-containing regimens at risk [11–13]. To overcome these limitations, next-generation diarylquinolines are being developed, including TBAJ-876. TBAJ-876 has become a promising drug candidate, as it has demonstrated more potent in vitro activity against *Mycobacterium tuberculosis*, as well as lower lipophilicity, a shorter half-life in mice, and a potential lower risk for cardiac events due to reduced hERG channel affinity compared to bedaquiline [9, 14]. Importantly, TBAJ-876 retains activity and is more potent than bedaquiline against *M. tuberculosis* with *Rv0678* mutations, the primary cause of bedaquiline resistance in clinical isolates [15].

Hence, interest has been growing in the preclinical activity of TBAJ-876 within various combination regimens [15, 16], and the compound recently entered the clinical trial phase. Currently, the results of 2 phase 1 clinical trials are awaited (NCT04493671 and NCT05526911), and a phase 2 trial is planned in which TBAJ-876 will replace bedaquiline within the BPaL regimen in the treatment of drug-sensitive tuberculosis (NCT06058299).

Preclinical studies play a crucial role in evaluating the activity of new drug candidates, providing valuable input for subsequent clinical trials design. For example, pharmacodynamic and pharmacokinetic mouse studies are used to estimate a drug's antituberculosis potency and contribute to adequate dose selection to achieve desirable effects in humans [17]. In this regard, diarylquinolines are of special interest because, in vivo, metabolites are formed that are also active against *M. tuberculosis* [9, 18]. Rouan et al demonstrated that for bedaquiline both the parent drug and its main M2 metabolite contributed significantly to the total bedaquiline activity observed in a tuberculosis mouse model [18]. However, in humans, it is anticipated that the metabolite's contribution is considerably less than in mice, due to lower exposures to the metabolite and the metabolite being less active against *M. tuberculosis* than the parent drug [18, 19]. Like bedaquiline, TBAJ-876 is metabolized in vivo, with TBAJ-876-M3 (M3) as the main metabolite, formed by *N*-demethylation [9, 16]. Taking into account metabolite activity and differences in parent-to-metabolite exposure ratios between mice and humans could improve animal model-based predictions on drug activity and drug exposure in humans.

The objective of this study was to determine the contribution of M3 to the overall bactericidal activity of TBAJ-876. First, the differential activity of TBAJ-876 and the M3 metabolite against actively replicating and nonreplicating *M. tuberculosis* was assessed in vitro. Subsequently, the in vivo bactericidal activity and pharmacokinetic properties of TBAJ-876 and M3 were determined in a mouse tuberculosis model. By integrating these findings, the contribution of the M3 metabolite to the overall bactericidal activity of TBAJ-876 in a mouse tuberculosis model was estimated.

## METHODS

### Bacterial Strains

In vitro experiments were performed with the *M. tuberculosis* H37Rv reference strain and/or the clinical *M. tuberculosis* Beijing VN 2002-1585 strain [20]. In the in vivo studies, *M. tuberculosis* Beijing VN 2002-1585 was used. Strains were grown in Middlebrook 7H9 broth (Becton, Dickinson, and Company [BD]) with 10% oleic acid, albumin, dextrose, catalase (OADC; BD) under shaking conditions at 37°C. Bacterial stock suspensions were stored at −80°C.

### In Vitro Activity Testing

Bedaquiline, TBAJ-876, and M3 were supplied by the TB Alliance. In vitro experiments were performed in duplicate. Bedaquiline was used as comparator. TBAJ-876 and M3 stock solutions were prepared in dimethyl sulfoxide. Bedaquiline was dissolved in acidified 20% hydroxypropyl-β-cyclodextrin (Kleptose; Roquette), followed by pH adjustment to 2.5 using 1 N sodium hydroxide. Minimum inhibitory concentrations (MICs) of TBAJ-876 (tested range, 0.063–0.001 mg/L), M3 (tested range, 0.002–0.125 mg/L), and bedaquiline (tested range, 0.008–0.5 mg/L) against *M. tuberculosis* H37Rv and Beijing VN 2002-1585 were determined by agar proportion based on the Clinical and Laboratory Standards Institute guidelines (M24, third edition) [21].

A checkerboard assay was conducted with *M. tuberculosis* Beijing VN 2002-1585 to assess potential interaction between TBAJ-876 and M3. Two-fold increasing concentrations of TBAJ-876 (0.0001–0.016 mg/L) and M3 (0.001–0.064 mg/L) in 7H9 with 10% OADC in the columns and rows of a 96-well plate, respectively, were inoculated with mycobacterial suspensions (5 × 10^5^ colony forming units [CFU]/mL). The MICs of TBAJ-876 and M3 alone and in combination were visually read after 10 days of incubation at 35°C with 5% CO_2_. The fractional inhibitory concentration (FIC) was calculated as the MIC of TBAJ-876 and M3 in combination divided by the MIC of TBAJ-876 and M3 alone. The FIC index was calculated as the FIC of TBAJ-876 plus the FIC of M3. An FIC index of ≤0.5 indicates synergy, > 4.0 antagonism, and 0.5–4.0 no interaction [22].

Time- and concentration-dependent activity of TBAJ-876 and M3 against actively replicating or nonreplicating *M. tuberculosis* H37Rv was determined as previously described [23]. Mycobacterial cultures were exposed to 4-fold increasing concentrations of TBAJ-876 (0.00025–0.063 mg/L), M3 (0.001–0.25 mg/L), and bedaquiline (0.004–1 mg/L). On days 1, 2, 3, and 6 culture samples were plated onto 7H10 agar plates to determine CFU/mL. A similar set-up was used to determine TBAJ-876 and M3 activity against nonreplicating mycobacteria. A nutrient starvation model was used to induce a nonreplicating state. Log-phase cultures were centrifuged for 20 minutes at 3000*g* and mycobacterial pellets were suspended in phosphate-buffered saline (PBS) with 0.05% tyloxapol. After a 6-week adaptation period at 37°C under shaking conditions, TBAJ-876 and M3 activity were tested as described above. The duplicate experiment was extended to 21 days.

### Ethical Approval Animal Studies

The studies involving animals (mice) were approved by the Erasmus MC Animal Welfare Body. All study protocols adhered to the rules specified in the Dutch Animal Experimentation Act and were in concordance with the European Union animal directive 2010/63/EU (license No. AVD1010020173687).

### Animals, Infection, and Treatment

Specified pathogen-free female BALB/c mice (Supplementary Material 1), aged 11–12 weeks at time of infection were obtained from Charles River. The mice were divided into 2 treatment groups: 48 animals per group treated with either TBAJ-876 or M3. Infection with *M. tuberculosis* Beijing VN 2002-1585 was as previously described [24]. Under general anesthesia, 1.06 × 10^5^ (range, 1.04–1.08 × 10^5^) CFUs were instilled intratracheally. The animals’ welfare was scored daily and the mice were euthanized in cases where humane end points (decreased response to stimuli, hunched posture, unkempt and dirty coat, respiratory distress) were reached.

TBAJ-876 and M3 were formulated weekly in 20% (wt/vol) hydroxypropyl-β-cyclodextrin (Kleptose; Roquette) acidified with 1.5% 1 N hydrogen chloride. After shaking overnight, the pH was raised to 2.0 by 1 N sodium hydroxide. Formulations were stored at 6°C. The desired dose in mg/kg was based on an average mouse body weight of 22 g.

Treatment was started 2 weeks after infection. In the TBAJ-876 group, animals were treated with 25, 6.3, or 1.6 mg/kg. In the M3 group, animals were treated with 50, 12.5, or 3.1 mg/kg. Higher doses were chosen for M3 considering the virulent character of the Beijing *M. tuberculosis* strain that was used for infection and the MIC of M3 being higher than that of TBAJ-876. TBAJ-876 and M3 were administered once daily via oral gavage (volume, 0.2 mL) for 5 days per week. Animal studies were performed in 2 separate experiments: one focusing on bactericidal activity and pharmacokinetics of TBAJ-876, the other on M3. Untreated animals were not included in this study for ethical reasons considering the virulent character of the *M. tuberculosis* strain, resulting in death within 2–3 weeks after infection without adequate treatment [25].

### Mycobacterial Load Assessment

Three animals were euthanized just before treatment initiation to determine the *M. tuberculosis* CFU/lung at baseline. Lungs were aseptically removed, homogenized in 2 mL PBS in M-tubes using the GentleMACS Octo Dissociator (Miltenyi Biotec), 10-fold serially diluted in PBS, and plated onto 7H10 agar. After 1, 2, or 3 weeks of treatment, 3 animals per treatment dose were euthanized to determine the CFU/lung as described above. To prevent drug carry-over, treatment was stopped 72 hours before euthanasia and lung homogenates were plated onto 7H10 agar containing activated charcoal (0.4%) and 10% OADC. Per 10-fold dilution, 200 µL of the solution was plated, giving a lower limit of detection of 11.5 CFU in the lungs, based on a total lung homogenate volume of 2.3 mL. The bactericidal activity was defined as the decrease in the CFU/lung compared to start of treatment.

### Pharmacokinetic Analyses

TBAJ-876 and M3 plasma concentrations were determined after 4 weeks of treatment, at steady state. Blood was collected by orbital sinus bleeding in ethylenediaminetetraacetic acid-containing microcentrifuge tubes, directly followed by euthanasia, at 1.5, 6, 24, and 96 hours (n = 2 mice per time point) after the last dose administration. Tubes were centrifuged (10 000*g*, 5 minutes) to obtain plasma. Acetonitrile was added in a plasma to acetonitrile ratio of 1:3, centrifuged (10 000*g*, 5 minutes), and clear supernatant was collected in cryotubes and frozen at −80°C. Per sample, 50 µL was plated onto 7H10 agar to confirm decontamination. Plasma samples were shipped on dry ice to Alliance Pharma (now Resolian, Malvern, Pennsylvania, USA) for analysis via liquid chromatography-tandem mass spectrometry (LC-MS/MS; Supplementary Material 2).

### Data Analysis

CFU counts were log_10_ transformed. The area under the plasma concentration-time curve (AUC_0-96_) was calculated by using the trapezoid rule. A nonlinear regression dose-response inhibition model was used to investigate the relationship between CFU/lung and M3 exposure following 4 weeks of treatment with TBAJ-876 or M3. Analyses were performed using Graphpad Prism 9.5.0 software (GraphPad Software).

## RESULTS

### In Vitro Studies: MIC, Checkerboard, and Time-Kill Kinetics Assays

**Figure 1.**
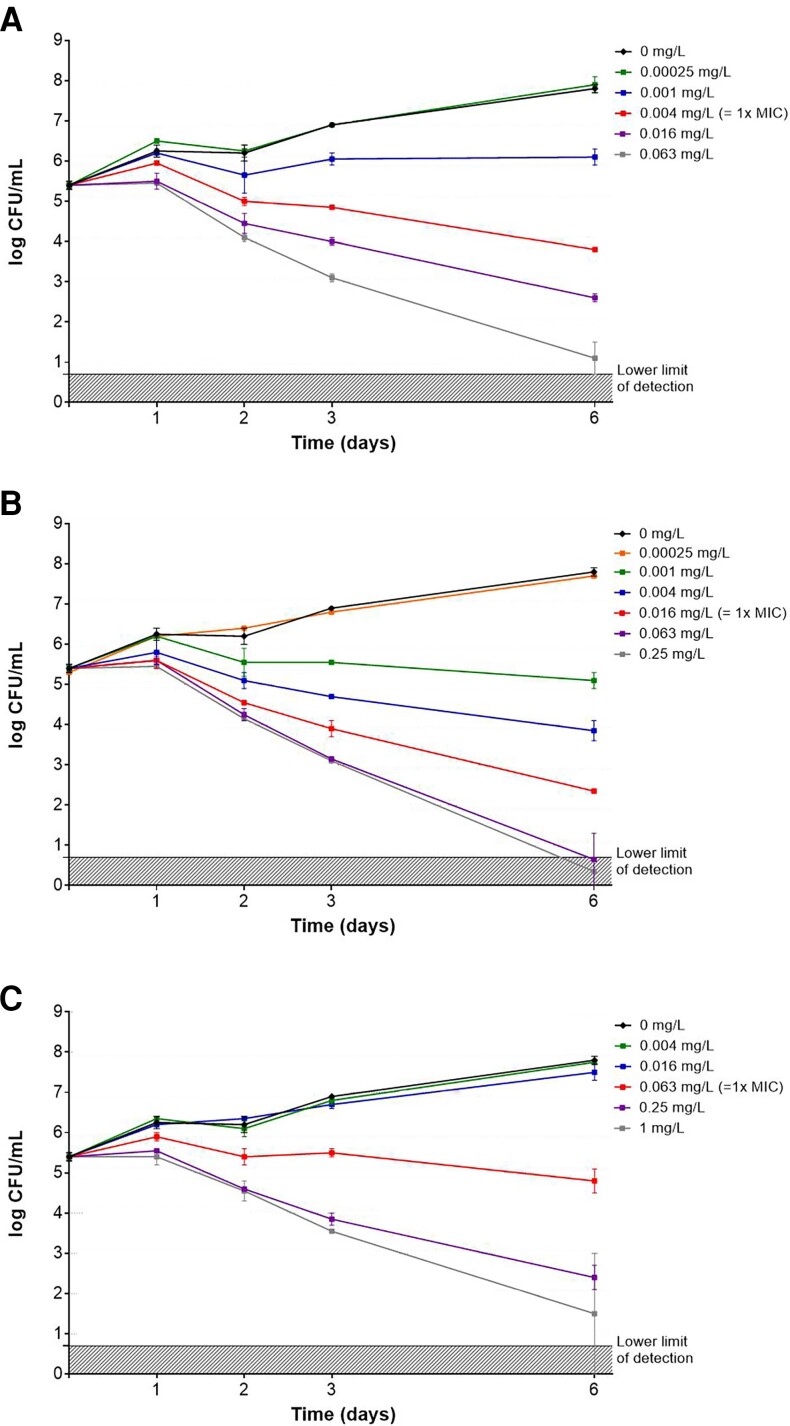
Time- and concentration-dependent activity of TBAJ-876 (*A*), M3 (*B*), and bedaquiline (*C*) against actively replicating *Mycobacterium tuberculosis* Beijing VN2002-1585. Mycobacterial cultures were exposed to 4-fold increasing concentrations. The experiment was performed in duplicate, and results are presented as mean log_10_ CFU/mL (± range). Abbreviations: CFU, colony-forming unit; MIC, minimum inhibitory concentration.

The aim of the in vitro assays was to determine the differential activity of TBAJ-876 and M3 against *M. tuberculosis*, with bedaquiline as comparator, including different bacterial metabolic states because mycobacteria can alter their metabolic activity upon external stresses encountered in vivo. MICs of TBAJ-876, M3, and bedaquiline against *M. tuberculosis* H37Rv and Beijing VN 2002-1585 are shown in Table 1. For all compounds, MICs for Beijing VN 2002-1585 were slightly lower than for H37Rv. MICs were 4-fold lower for TBAJ-876 than M3. The MIC of TBAJ-876 was 8 to 32-fold lower than that of bedaquiline. The checkerboard assay demonstrated a FIC index range of 0.51–2.25, indicating a lack of interaction between TBAJ-876 and M3 against Beijing VN2002-1585. Against actively replicating *M. tuberculosis*, time- and concentration-dependent activity of TBAJ-876 and M3 was similar, whereas bedaquiline required higher concentrations to achieve equivalent activity (Figure 1). Substantially reduced and delayed killing activity was observed against nonreplicating *M. tuberculosis* for TBAJ-876, M3, and bedaquiline (Supplementary Figure 1). Only the highest concentration (16 × MIC) of TBAJ-876 and M3 exhibited activity, with 3 weeks of exposure leading to a decrease of approximately 1.6 log_10_ CFU/mL compared to the no drug control.

**Table 1.** Minimum Inhibitory Concentrations of TBAJ-876, M3, and Bedaquiline Against *Mycobacterium tuberculosis* H37Rv and *M. tuberculosis* Beijing VN2002-1585

Strain	Minimum Inhibitory Concentration (mg/L)		
	TBAJ-876	M3	Bedaquiline
*M. tuberculosis* H37Rv	0.008–0.016	0.031–0.063	0.125
*M. tuberculosis* Beijing VN 2002-1585	0.004	0.016	0.063–0.125

### In Vivo Bactericidal Activity and Pharmacokinetics

**Figure 2.**
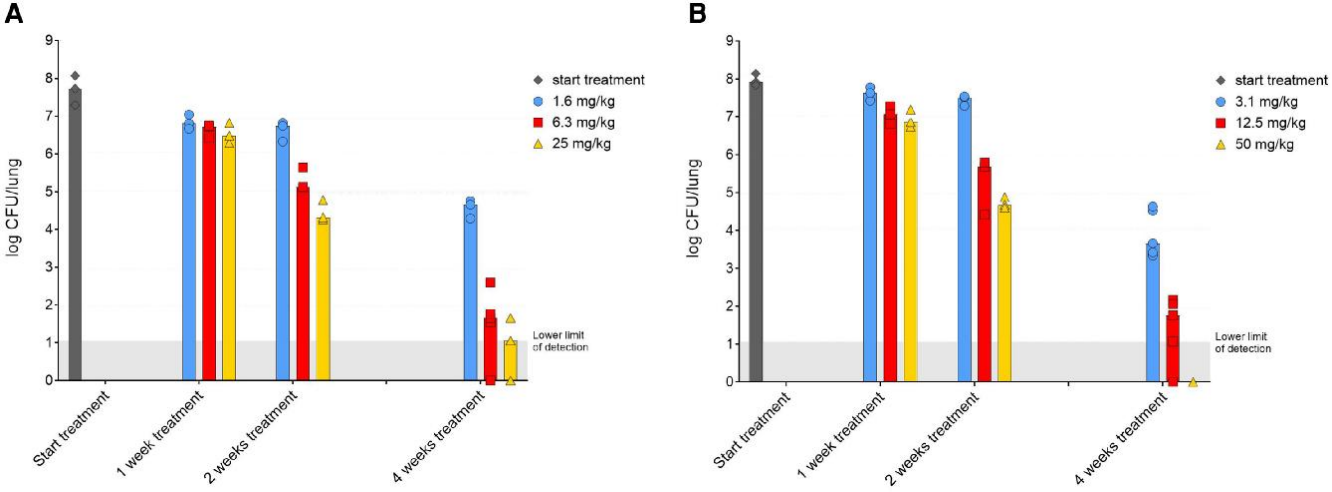
In vivo bactericidal activity of TBAJ-876 (*A*) and M3 (*B*) against *Mycobacterium tuberculosis* Beijing VN2002-1585. The mycobacterial load is expressed as log_10_ colony-forming units (CFU) in the lungs after 0, 1, 2, or 4 weeks of treatment. TBAJ-876 was dosed at 1.6, 6.3, or 25 mg/kg. M3 was dosed at 3.1, 12.5, or 50 mg/kg. Three animals were included per dose and treatment duration. Two extra animals were added to the groups that were treated for 4 weeks (n = 5 per dose), which were animals included in the study for plasma concentration measurement at 96 hours after the last dose administration. One animal in the M3 experiment reached humane end points due to complications of the intratracheal *M. tuberculosis* instillation resulting in n = 4 in the 50 mg/kg 4 weeks’ treatment group.

TBAJ-876 was well tolerated by the mice at the tested doses. However, the animals treated with 50 mg/kg M3 continued to lose weight throughout the experiment while the tuberculosis infection was adequately controlled, indicating possible adverse effects of the metabolite administration. Both TBAJ-876 and M3 were bactericidal in a dose- and time-dependent manner (Figure 2). After 4 weeks of treatment with TBAJ-876, the median CFU count in the lungs declined from 7.7 log_10_ CFU at treatment initiation to 4.3, 1.7 (1/5 animal was culture negative), and 1.1 log_10_ (2/5 culture negative) in animals treated with 1.6, 6.3, or 25 mg/kg, respectively. The median CFU count in the lungs of animals treated for 4 weeks with M3 declined from 7.9 log_10_ CFU to 3.7 (0/5 culture negative), 1.8 (1/5 culture-negative), and 0 log_10_ CFU (4/4 culture negative) when dosed at 3.1, 12.5, or 50 mg/kg, respectively.

**Figure 3.**
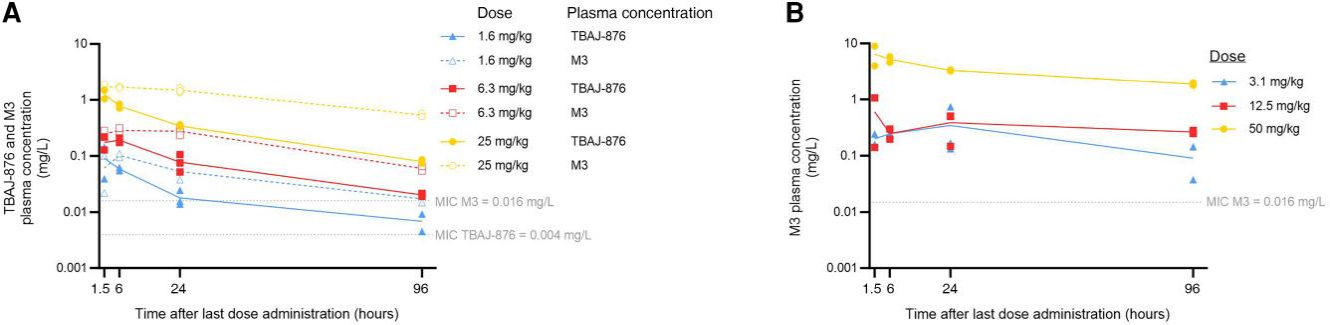
In vivo plasma concentrations of TBAJ-876 and M3 in animals infected with *Mycobacterium tuberculosis* Beijing VN2002-1585 and treated with TBAJ-876 dosed at 1.6, 6.3, or 25 mg/kg (*A*) or M3 dosed at 3.1, 12.5, or 50 mg/kg (*B*) for 4 weeks. Each plasma concentration measurement is plotted individually. Two animals were included per time point. To all 24-hour groups, except for M3 50 mg/kg, 1 additional animal was included, which were spare animals that were included in case of drop-outs. In TBAJ-876–treated animals, plasma concentrations of both TBAJ-876 (solid lines) and M3 (dashed lines) were determined. In M3-treated animals, plasma concentrations of M3 were measured. The horizontal dotted lines represent the minimum inhibitory concentration (MIC) of TBAJ-876 and M3.

Steady-state plasma concentration-time profiles of TBAJ-876 and M3 after 4 weeks of TBAJ-876 treatment were dose dependent (Figure 3*A*). Following TBAJ-876 treatment, exposure (AUC_0-96_ mg·h/L) to M3 was 2.2 to 3.6-fold higher than exposure to TBAJ-876 (Table 2). In M3-treated animals, dose-dependent plasma concentration-time profiles were also observed, although the dose of 12.5 mg/kg resulted in relatively low M3 plasma concentrations and exposure (Figure 3*B* and Table 2).

**Table 2.** *Mycobacterium tuberculosis* CFU in the Lungs in Relation to Compound Exposure

Dose, mg/kg	Median log_10_ CFU/Lung at Start of Treatment	Median log_10_ CFU/Lung After 4 wk of Treatment	TBAJ-876 AUC_0-96_, mg·h/L	M3 AUC_0-96_, mg·h/L
TBAJ-876				
1.6	7.7	4.3	1.9	4.3
6.3	7.7	1.7	6.8	18.4
25	7.7	1.1	29.9	108.8
M3				
3.1	7.9	3.7	…	22.1
12.5	7.9	1.8	…	33.1
50	7.9	0.0	…	290.9

Abbreviations: AUC, area under the curve; CFU, colony-forming unit.

**Figure 4.**
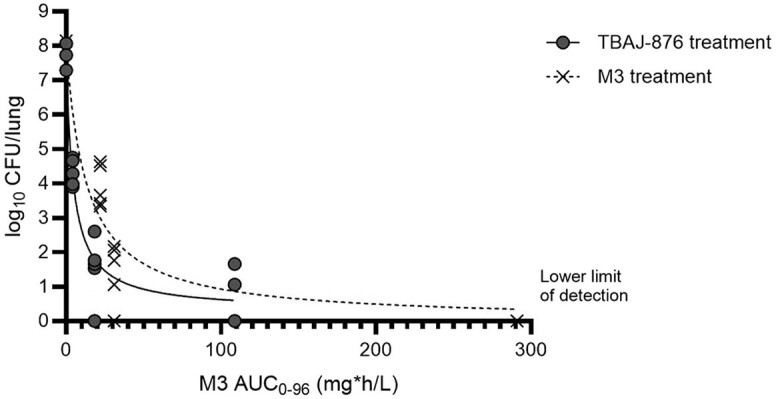
*Mycobacterium tuberculosis* colony-forming units (CFU) in the lungs in relation to M3 exposure, defined as area under the plasma concentration time-curve from 0 to 96 hours (AUC_0-96_). Lung CFU count was plotted against exposure to M3 after treatment with TBAJ-876 (circles) or M3 (crosses). The lines represent the relationship between lung CFU count and exposure to M3 after TBAJ-876 treatment (solid line), or M3 treatment (dashed line), based on a nonlinear regression dose-response inhibition model. The lung CFU count at x = 0 aligns with the lung CFU count at the start of treatment, as no untreated animals were included in this study.

Figure 4 shows the lung CFU count relative to the exposure to M3 after treatment with either TBAJ-876 or M3. By comparing lung CFU counts at a specific level of M3 exposure following TBAJ-876 treatment to lung CFU counts at the same level of M3 exposure following M3 treatment, the relative contribution of M3 to the overall activity observed after TBAJ-876 treatment can be estimated. The underlying principle is that TBAJ-876 treatment leads to both TBAJ-876 and M3 exposure, which both determine the overall bactericidal activity, assuming no interactions between TBAJ-876 and M3 (as indicated by the checkerboard assay results). The levels of M3 exposure measured after TBAJ-876 treatment were associated with pronounced bactericidal activity in animals treated with M3 directly. For example, TBAJ-876 treatment at 6.3 mg/kg led to an M3 AUC_0-96_ of 18.4 mg·h/L and resulted in a CFU decline of 7.7 to 1.7 log_10_. M3 dosing at 3.1 mg/kg produced similar M3 exposures (22.1 mg·h/L), and lowered the lung CFU counts from 7.9 to 3.7 log_10_ (Table 2 and  Figure 4). This indicates that, within this mouse tuberculosis model, the M3 metabolite contributes substantially to the overall bactericidal activity observed after TBAJ-876 treatment.

## DISCUSSION

This study investigated the relative contribution of the main TBAJ-876 metabolite, M3, to the overall activity of TBAJ-876. So far, TBAJ-876 activity has been evaluated in several preclinical studies [9, 15, 16, 26]. However, this is the first study to assess its activity while considering the pharmacokinetics and direct bactericidal activity of the M3 metabolite. Furthermore, previous studies were conducted with *M. tuberculosis* H37Rv, whereas this study used a clinical strain of the Beijing genotype, known for its virulent properties [24, 27, 28].

A recent mouse study investigated the pharmacokinetic properties of TBAJ-876 and M3 in uninfected mice after 1 or 7 days of TBAJ-876 treatment at 3.1 or 6.3 mg/kg [16]. Similar to our findings, the authors reported that M3 plasma concentrations were higher than TBAJ-876 following TBAJ-876 treatment, the AUC of M3 being 2.4 to 4.1-fold higher than that of TBAJ-876.

Another study by Almeida et al. evaluated the bactericidal activity of TBAJ-876 dosed at 3.1, 6.3, or 12.5 mg/kg against *M. tuberculosis* H37Rv in a comparable mouse tuberculosis model [15]. Four weeks’ treatment lowered lung CFU counts from 7.6 log_10_ at the start of treatment to 4.4, 3.2, and 2.4 log_10_ CFU for the 3 different doses, respectively. Our study confirms the profound bactericidal activity of TBAJ-876. In fact, TBAJ-876 activity was more pronounced here. This might be attributable to differences in experimental design, including the strain used for infection. Almeida et al used the H37Rv strain, whereas we used a Beijing strain [15]. While subtle (2 to 4-fold difference), the MIC discrepancies observed between the 2 strains could hint at a possibility of higher TBAJ-876 activity against Beijing strains compared to H37Rv. Consistent with other studies [9, 15, 29], TBAJ-876 seems more potent than bedaquiline, both dose based and exposure based [15, 29].

Mouse tuberculosis models are an important part of the drug development pipeline. They provide insight into the in vivo antituberculosis activity of drug candidates within the complex system of an infected host, and enable prioritization of drug candidates and drug combinations before advancing to human clinical studies. Additionally, mouse studies can provide data for translating and predicting the dosage necessary to achieve desired treatment outcomes in humans. However, in such studies, the potential antituberculosis activity of in vivo formed metabolites is often not considered. Acknowledging the significance of metabolite activity and variations in metabolism between mice and humans can provide insight into whether drug activity in mice might be underestimated or overestimated compared to humans, and, as such, can enhance the accuracy of animal model-based predictions (both direct or via predictive models using preclinical data) regarding drug activity and drug exposure in humans [17, 30].

For TBAJ-876 and M3, the parent-to-metabolite ratio in humans is not yet reported, but results are pending (NCT04493671 and NCT05526911). In direct opposition to observations in mice, where exposures to bedaquiline's main metabolite, *N-*desmethyl bedaquiline, are 2.6 to 3.3-fold higher than the parent drug, in humans, the metabolite exposure is 4 to 5-fold lower compared to the parent drug [18, 19], suggesting a comparable scenario for TBAJ-876. As such, it is relevant to study M3's contribution to the total TBAJ-876 activity by investigating the individual activity of both compounds and the parent-to-metabolite ratio, aiming to increase the predictive value of preclinical models.

Species-specific differences in parent-to-metabolite ratios are especially relevant when metabolites are formed with distinct activity patterns against *M. tuberculosis* compared to the parent drug. Assessing the individual activity of TBAJ-876 and M3 in a mouse tuberculosis model following TBAJ-876 treatment poses a hurdle due to the concomitant formation of M3. Two approaches were used to overcome this challenge: using in vitro studies to compare TBAJ-876 and M3 activity and direct administration of the M3 metabolite to the animals in the mouse tuberculosis model. Evaluation of in vitro activity against nonreplicating *M. tuberculosis* was included because mycobacteria can alter their metabolic level in response to diverse stresses encountered within the microenvironment of tuberculosis lesions, including limited nutrient availability [31]. When parent drug and metabolite demonstrate distinct activity patterns against *M. tuberculosis* in different metabolic state, acknowledging species-specific parent-to-metabolite ratios might be of particular importance considering differential pathology in mice versus humans [30, 32]. Regarding the MIC, a 4-fold difference was observed between TBAJ-876 and M3, comparable to the MIC difference between bedaquiline and its M2 metabolite [18]. However, the time- and concentration-dependent activity against actively replicating and nonreplicating *M. tuberculosis* was similar for TBAJ-876 and M3. In vivo, M3 bactericidal activity was potent, with the highest concentration rendering the lungs of all mice culture negative after only 4 weeks of treatment. Given the 2.2 to 3.6-fold higher exposure to M3 than to TBAJ-876 following treatment with TBAJ-876, combined with the profound bactericidal activity of M3, the total activity seen upon TBAJ-876 treatment seemed to be substantially attributable to M3 in our mouse model.

This study focused on variation in bactericidal activity between parent and metabolite and their relative contribution to the overall activity. In the context of (active) metabolite formation, additional factors apart from variation in bactericidal activity could be important when extrapolating findings from mouse models to humans. For example, studying whether parent and metabolite are equipotent in penetrating different regions of tuberculosis pulmonary lesions or whether they differ in activity against intracellular bacilli can be relevant [30, 33], especially because BALB/c mice develop cellular granulomas [32], whereas human tuberculosis granulomas are often necrotizing and caseous in nature. Even more importantly, in light of species-specific parent-to-metabolite ratios, differences in toxicity profiles of parent and metabolite could impact the association between safety of a drug in animal studies and what is expected in clinical practice [34].

Although this study design allows for an estimation of role of the main metabolite within the total drug activity based on in vitro data and in vivo early bactericidal activity, the modest scale and exploratory nature of this study should be considered. The pharmacokinetics of TBAJ-876 and M3 relies on a limited dataset, containing 4 time points after the last dose administration with up to 3 animals per time point. However, because plasma concentration-time profiles of TBAJ-876 and M3 are reported as relatively static [16], the restricted number of time points is unlikely to have considerably impacted the estimation of compound exposure based on AUC_0-96_. However, exposure to M3 following M3 treatment at 12.5 mg/kg was not proportional with the lower and higher doses tested, and additional sampling time points or larger group sizes could have provided a more comprehensive understanding of whether the observed M3 exposure was underestimated.

In conclusion, TBAJ-876 and M3 demonstrated profound activity against *M. tuberculosis* both in vitro and in the mouse tuberculosis model. Notably, the in vivo activity of TBAJ-876 treatment appeared to depend largely on M3, given its relatively high exposure compared to the parent compound, together with its potent activity. Based on the similar potency of TBAJ-876 and M3, clinical activity of TBAJ-876 can be anticipated regardless of the parent-to-metabolite ratio, provided that exposures required for activity can be reached safely in humans. While potential species-specific differences in parent-to-metabolite ratios for TBAJ-876 and M3 may not limit extrapolation of mouse models results to humans, we advocate taking metabolites and their potentially distinct activity profiles into account to enhance the accuracy of mouse model-derived predictions on treatment efficacy in humans.

## Supplementary Data

Supplementary materials are available at *The Journal of Infectious Diseases* online (http://jid.oxfordjournals.org/). Supplementary materials consist of data provided by the author that are published to benefit the reader. The posted materials are not copyedited. The contents of all supplementary data are the sole responsibility of the authors. Questions or messages regarding errors should be addressed to the author.

## Supplementary Material

jiae332_Supplementary_Data

## Notes

***Acknowledgments***. The authors thank Aart van der Meijden, Heleen van der Spek, and Sanne van den Berg for their technical assistance; and the animal biosafety laboratory personnel for their biotechnical and functional support.

***Author contributions.*** S. E. M. contributed conceptualization, methodology, formal analysis, investigation, writing the original draft, visualization, and project administration. N. C. A. contributed conceptualization, methodology, investigation, and writing review and editing. M. T. K. contributed investigation, methodology, and writing review and editing. N. F. contributed conceptualization, resources, and writing review and editing. M. U. L. contributed resources, and writing review and editing. H. I. B. contributed conceptualization, methodology, and writing review and editing. J. E. M. S. contributed conceptualization, methodology, writing review and editing, supervision, project administration, and funding acquisition.

***Funding*.** This work was supported by the TB Alliance with support from Australia's Department of Foreign Affairs and Trade, the UK Foreign, Commonwealth & Development Office, Bill and Melinda Gates Foundation (OPP1129600), and German Federal Ministry of Education and Research.
