# Supplementary material for: Relative Contributions of the Novel Diarylquinoline TBAJ-876 and its Active Metabolite to the Bactericidal Activity in a Murine Model of Tuberculosis
Source: J Infect Dis. 2024 Jun 28;230(6):e1366–74. doi: 10.1093/infdis/jiae332 (PMC11646583; doi:10.1093/infdis/jiae332)
Supplement: jiae332_Supplementary_Data [file jiae332_supplementary_data.docx]

**Relative contributions of the novel diarylquinoline TBAJ-876 and its active metabolite to the bactericidal activity in a murine model of tuberculosis**

Saskia E. Mudde, Nicole C. Ammerman, Marian T. ten Kate, Nader Fotouhi, Manisha U. Lotlikar, Hannelore I. Bax, Jurriaan E.M. de Steenwinkel

**Online Supplementary files**

**Supplementary file 1: female BALB/c mice**

This study was performed with female mice only, as they can be housed together, whereas this is not preferable for male mice. Since mice are social animals, individual housing would lead to increased stress levels. Considering the length of these experiments, the additional stress of individual housing is deemed disproportionate to the potential added scientific value. Furthermore, although sex could potentially influence drug exposure after treatment, this study focuses on the relation between drug exposure and drug activity, which is generally not influenced by sex.

**Supplementary file 2: Material and methods pharmacokinetic analysis by LC-MS/MS**

Neat stock solutions for TBAJ-876 and M3 were prepared in DMSO at 1 mg/mL. A standard curve ranging from 1 ng/mL to 1,000 ng/mL was prepared in mouse plasma (BioIVT). For quality control, 3 concentrations were tested: 3, 50, and 800 ng/mL. The calculated concentrations were deemed acceptable if the accuracy of the quality control samples was ±20% of the nominal concentrations. The lower limit of quantification in plasma was 4 ng/mL. The blank matrix, standards, and quality control samples were prepared in a 1:3 ACN ratio to parallel the study samples. For these samples and the study samples, 10 µl was transferred to clean tubes on wet ice, followed by addition of 50 µl acetonitrile (ACN) per tube. As internal standard, 100 µl of 100 ng/mL of TBAJ-876-d6 with 0.1% formic acid in ACN was added to all tubes. The samples were thoroughly vortexed, followed by centrifugation at 20,000xg for 3 minutes at room temperature. For each sample, 50 µl of the supernatant was transferred to a clean Axygen 96-well collection plate containing 150 µl of 0.1% formic acid in ACN:water (20:80). The plate was vortexed and centrifuged at 1,670xg for 5 minutes at room temperature. The mixtures were either stored at 2-8°C or directly analysed by HPLC set at 4°C. For the plasma concentration analysis an Agilent 1290 Infinity II high-performance liquid chromatography system with Acquity BEH C18 column at room temperature (2.1 x 50 mm, 1.7 µm) and an Agilent 6495C MS/MS system were used. LC was performed with ammonium acetate 10 mM and 0.1% formic acid in water for mobile phase A, and 0.1% formic acid in ACN for phase B. The injection volume was 5 µl with a flow of 0.60 mL/min. Following electrospray ionization, analytes were quantified by multiple-reaction monitoring (MRM), using the following MRM transitions (m/z): TBAJ-876 (657.1/239.1); TBAJ-876-M3 (643.2/568.2); TBAJ-876-d6 (663.0/245.1). Data were processed using MassHunter Data Acquisition software, version 10.1.

**
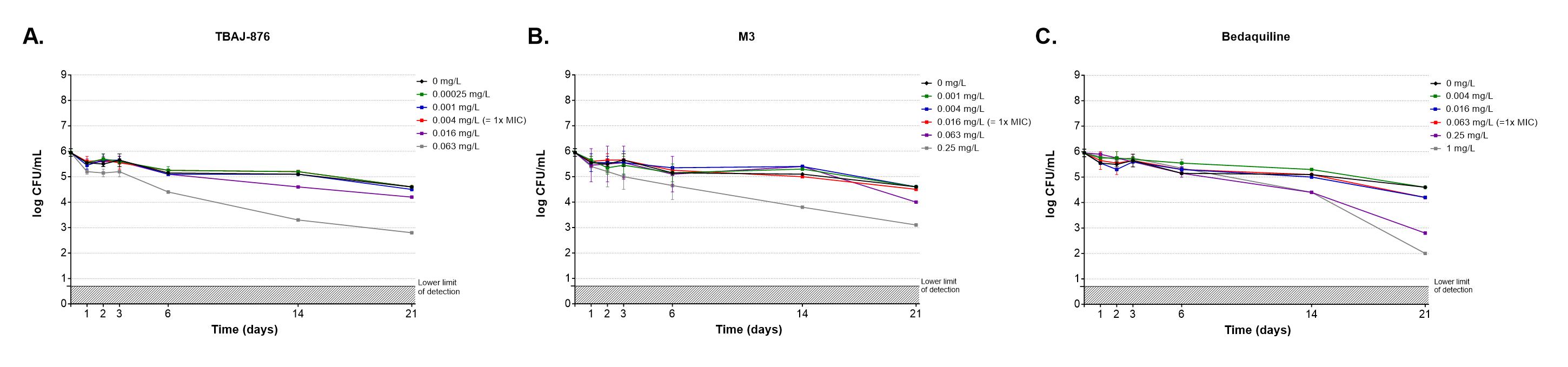
**

**Figure S1.** Time- and concentration dependent activity of TBAJ-876, M3, and bedaquiline against non-replicating *M. tuberculosis* Beijing VN2002-1585. The non-replicating state was induced by nutrient starvation. Cultures were exposed to four-fold increasing concentrations. The experiment was performed in duplicate, except for sampling days 14 and 21. Results are presented as mean log_10_ *M. tuberculosis* colony-forming units (CFU) per mL (+/- the range).
